# Good clinical practice regulatory inspections: Lessons for Indian investigator sites

**DOI:** 10.4103/2229-3485.71776

**Published:** 2010

**Authors:** R. Marwah, K. Van de Voorde, J. Parchman

**Affiliations:** *Department of Clinical Trial and Safety, Bristol-Myers Squibb Company, Global Clinical Research and Regulatory Compliance*

**Keywords:** Corrective and preventive action plan, compliance, findings, inspections, warning letter

## Abstract

Regulatory inspections are important to evaluate the integrity of the data submitted to health authorities (HAs), protect patient safety, and assess adequacy of site/sponsor quality systems to achieve the same. Inspections generally occur after submission of data for marketing approval of an investigational drug. In recent years, there has been a significant increase in number of inspections by different HAs, including in India. The assessors/inspectors generally do a thorough review of site data before inspections. All aspects of ICH-GCP, site infrastructure, and quality control systems are assessed during the inspection. Findings are discussed during the close out meeting and a detailed inspection report issued afterward, which has to be responded to within 15–30 days with effective Corrective and Preventive Action Plan (CAPA). Protocol noncompliance, inadequate/inaccurate records, inadequate drug accountability, informed consent issues, and adverse event reporting were some of the most common findings observed during recent Food and Drug Administration (FDA) inspections. Drug development is being increasingly globalized and an increased number of patients enrolled in studies submitted as part of applications come from all over the world including India. Because of the steep increase in research activity in the country, inexperienced sites, and more stakeholders, increased efforts will be required to ensure continuous quality and compliance. HAs have also made clear that enforcement will be increased and be swift, aggressive, and effective.

## INTRODUCTION

Regulatory inspections are an important and essential part of clinical research to evaluate the integrity of the data submitted to health authorities (HAs), presence of infrastructure to conduct clinical research, measures implemented to protect patient’s interest and safety, adequacy of site/sponsor quality systems, and verification of compliance with the principles of ICH-GCP as well as local regulations.

Inspections generally occur after submission of data for marketing approval of an investigational drug; however, inspections may happen at any time during the conduct of the trial like FDA’s Early Intervention Program.

All HAs like US FDA, EMA (European Medicines Agency) and others to whom data have been submitted from Indian site(s) may conduct inspections at the respective study sites.

Drug Controller General India (DCGI) has jurisdiction to inspect all clinical trials. DCGI has conducted inspections to investigate issues, where required. A routine inspection program will also start in near future.

A closer look at recent inspection frequency figures suggests a significant number of inspections occurring outside the US including in the Asia Pacific region. In fiscal year 2009, FDA Center for Drug Evaluation Research (CDER) clinical investigator inspections totaled 458 and 119 of those (26%) were outside the US. The FDA Clinical Investigator Inspection List (CLIL) website, an online posting of inspections with final classifications, shows 78 investigator site inspections in the Asia-Pac region, out of which 14 (6 in 2009) have been in India.[1]

The results have been positive with either “No Action Indicated” or “Voluntary action Indicated.” The findings were in categories of Drug Accountability, Protocol Compliance, Inaccurate Records, and Failure to follow Investigational Plan.

Because of the steep increase in research activity, inexperienced sites, and more stakeholders, a lot of effort will be required to ensure continuous quality and compliance to maintain this positive trend in inspection outcomes.

EMA has also increased inspections in the region with India being one of the most inspected countries (eight inspections between 1997 and 2008) outside the EU (European Union). This is in keeping with the increased number of subjects recruited from the region (11.6% in pivotal studies submitted in 2007 versus 4% for submissions between 2005 and 2007) for the pivotal studies submitted to EMA for approval.[2]

## PREINSPECTION ACTIVITIES

The HAs generally contact sponsors to arrange for site inspections and confirmation of dates. Subsequently, there may be direct communication between HAs and sites. Inspectors may request for preinspection documents such as curriculum vitae of (sub) investigators, serious adverse events (SAE), and also documentation of sponsor oversight, such as monitoring reports. It should be noted that site data (as submitted in marketing approval package) would generally have been thoroughly reviewed by assessors, inspectors, and other subject matter experts before inspection. Therefore inspectors may come prepared with specific queries about safety and efficacy data, GCP compliance issues, and/or other points.

The sponsor role is to coordinate between inspectors and site personnel, ensure that all documents are present/made available during inspection, all internal subject matter experts have been informed to be available for any queries/document requests, site personnel are available, appointments have been made for facility tour/queries with all departments involved in study (e.g. Labs, Pharmacy, Radiology, Archival, etc.), and logistics have been taken care of. Usually sites need to sign a statement to confirm that access to patient files will be granted.

## INSPECTION PROCESS

Generally, inspections are conducted by 1–2 inspectors and may last for 1 week depending on the study.

All site personnel who had a significant role in the conduct of trial should be present during the inspection – principal investigator (PI), sub-investigators (SI), Study coordinator (SC), radiologist, lab personnel, pharmacist (as applicable).

Sponsor representatives may be present at the site to facilitate the process; however, it should be clearly understood by all personnel that it is site inspection and site personnel should be at the forefront. Undue interference by sponsors may not be taken well by the inspectors.

The inspection starts with an opening meeting where introductions are made, purpose of inspection is discussed. The PI/representative makes a general presentation about the site, study, and addresses initial questions from inspectors. It is an appropriate time to discuss the inspection schedule, availability of site personnel, logistic arrangements, etc. to ensure smooth conduct of inspection with minimum interference in routine site schedule. If there were important deviations from GCP, it might be wise to be upfront with the inspectors and explain why they happened and how measures were taken to prevent them from happening again.

The inspection generally involves a facility tour to evaluate site infrastructure, site’s (written) standard operation procedures, and processes for their adequacy to conduct clinical trials. It may include: labs (if study related lab tests were done locally), review of lab equipments, process, calibrations, quality control methods, accreditation documents, training and handling of study specific lab queries; radiology/other equipments used in study, their calibration, maintenance schedule; archival-storage area for documents, the security, pest, humidity, and fire controls; investigational drug storage-accessibility, security, storage conditions, temperature records, thermometer calibrations, stock checks, labeling, accountability logs; out-patient department, clinical trial facility (as applicable).

The inspection focus is on investigator role in the study, delegation of duties (with documentation), qualification of site staff, ethical issues – consent process, and ethic committee review.

The data review generally includes subject history, informed consent, subject eligibility, investigational drug administration, compliance, safety reporting, compliance to study procedures, and other study-specific issues. There is a frequent interaction between inspectors and site personnel to clarify points, issues, and to provide supporting documents.

The inspection also involves team member interviews – PI, SI, SC, Lab personnel, others. Sponsor/monitor may also be interviewed to evaluate their supervision, protocol knowledge, adequacy to identify, and resolve issues.

The inspectors may also focus on insurance coverage for subjects, disaster plan in case of an emergency like fire, flood among other points.

At the end, there is exit meeting where findings are shared with site and sponsor personnel. Only if the investigator agrees, the sponsor may attend the close-out meeting. This also provides opportunity for site personnel to clarify any points concerning the findings or to provide better context.

## COMMUNICATION OF RESULTS

FDA may issue a list of findings referred to as FD483, which is handed over to the principal investigator at the end of the inspection. A detailed inspection report is issued to the site and inspection results are entered into FDA database. The findings are classified into: NAI (no action indicated), no objectionable conditions or practices were found during the inspection; VAI (voluntary action indicated), objectionable conditions were found but the problems do not justify further regulatory action and any corrective action is left to the investigator to take voluntarily; OAI (official action indicated), objectionable conditions were found and regulatory and/or administrative sanctions by FDA are indicated.

While not all FDA inspections require a written response, in general practice, the site/sponsor will review the findings, analyze the root cause, and submit an effective CAPA plan to FDA within 15 days.

The reporting inspector on behalf of EMA also issues a report of the main findings to the company inspected. The findings are classified into critical, major, and minor. The site and sponsor have to provide response/remedial actions within 15 days.

Some national HAs may give 30 days timeline for response.

## TIPS FOR A SUCCESSFUL INSPECTION

Some tips that may help to ensure a smooth conduct of an inspection are – to prepare for inspection from Day 1, i.e., always be GCP and regulations compliant; know your protocol and study requirements well; organize documentation well and provide accurate, complete, final version of documents requested; have key personnel/subject matter experts available to respond to questions; answer questions only in your area of responsibility; request clarification on any question or issue that is not clear, answer only the question asked and be upfront if you don’t know the answer, if follow-up is needed in order to respond to question, follow-up immediately; provide privacy to inspection team, but periodically check on progress and ask if clarification is needed; keep track of requested documents and provide them at the earliest; prepare overview of study conduct/roles and responsibilities of staff; maintain respectful, professional, and cooperative demeanor.

## COMMON FINDINGS

Protocol noncompliance, inadequate/inaccurate records, inadequate drug accountability, informed consent issues, and adverse event reporting were some of the most common findings observed during recent FDA inspections.

There has been an increase in the issuance of warning letters to clinical investigators by FDA (3 in 2005 to 23 in 2009).[3] Also, inadequate CAPA plans are being rejected by the HA. In this regard, it may be helpful to read a recent warning letter posted at FDA’s website: http://www.fda.gov/foi/warning_letters/s7104c.htm

This warning letter clearly demonstrates that GCP violations are unacceptable; in case they occur, immediate and effective actions are expected; principal investigator is responsible for site’s compliance to GCP norms and will be held accountable for noncompliance, lack of or delayed action plans to resolve and prevent the issues may result in further actions by FDA. In addition, the sponsor may be criticized for not having identified the issues and not taking actions, such as holding further patient enrolment till the issues are resolved or have improved.

## CITATIONS FROM WARNING LETTERS 2008–09[3]

Some of the common issues from recent FDA warning letters are cited here. It may benefit sites/sponsors to understand these issues, check if they exist in their own studies and take effective actions now to correct and prevent them.

- Inadequate human subject protection. Issues include nondisclosure of right to refuse to participate in study, denial of withdrawal from study, repeated or deliberate failure to provide study information in language understandable to subject or legally authorized representative; failure to appropriately delegate duties to qualified personnel (e.g., physical exams, SAE evaluation) with resultant exposure of subjects to unreasonable and significant risk or injury, subjects started on treatment before checking safety/baseline reports; failure to assure that the study has Institutional Review Board (IRB) review/approval; failure to assure that IRB has reviewed/approved changes in research; enrollment of subjects before IRB approval; subjects not consented before enrolment, not (timely) reconsented on updated safety information; enrollment of ineligible subjects; failure to (timely) report serious or life-threatening events to sponsor.
- Submission of false information, fabricated/altered/concealed study records to FDA or the sponsor; missing records/evidence records that have been deliberately destroyed or discarded; information changed after the subject had completed the study up to 2 years postcompletion;
- Repeated or deliberate failure to comply with regulations. Administration of study drug to subjects not authorized to receive it; subjects overdosed due to lack of dose adjustments based on weight/toxicity; no or inadequate records of receipt, preparation, use and/or disposition of study drug; records of study medication storage conditions not maintained to confirm the integrity of the investigational product; use of prohibited concomitant medications; numerous inaccuracies noted in the source documents and CRFs such that integrity of data collected at site cannot be verified.

In case FDA believes that it has evidence that the clinical investigator repeatedly or deliberately violated FDA’s regulations governing the proper conduct of clinical studies involving investigational products or submitted false information to the sponsor, it may issue initiate proceedings to restrict or disqualify the investigator from participating in clinical research.

## THE FUTURE

FDA has made clear that enforcement will be increased and be swift, aggressive, and effective.[4] Companies are expected to strictly adhere to legal requirements designed to protect product quality and integrity. All parties involved in the drug lifecycle must recognize that they have an ethical liability.

The EMA Work program 2009 and 2010 both stated that there will be a greater supervision of the conduct and ethical standards of clinical trials performed outside the EU and used in connection with marketing authorization applications in the EU. The number of GCP inspections was increased substantially in 2007 and remained at similar level since around 60 per year. Of note is that there were as many inspections conducted in third countries as in Europe itself, 19 in 2008.[5]

There is also an intent of contribution to capacity building with developing countries in cooperation with Member States and European Commission initiatives as inspections in the Rest of World (ROW) region are mainly dependent on FDA and EU activities. It is, therefore, important that local supervision in every country is supported and strengthened through capacity building, networking, information exchange, and by taking advantage of opportunities for joint or observed inspections. In September 2009, FDA and EMA started a pilot to collaborate on inspections in third countries.[6]

The EMA published a reflection paper in October 2009 on the extrapolation of results from clinical studies conducted outside the EU to the EU population.[6] They are further developing guidance on ethical and data quality requirements and on the assessment, inspection, and transparency processes related to clinical trials conducted in third countries.[7] They will also prepare a document with specific triggers for assessors in the context of the assessment of acceptability of clinical trials from third countries.

## SUMMARY OF HEALTH AUTHORITY EXPECTATIONS

Some of the expectations from HAs are as follows:

- Infrastructure – a well-trained, experienced, qualified research team, facility, processes to handle all research-related activities with back-up arrangement, principal investigator’s accountability/oversight for study conduct;
- Good medical records, documentation practice;
- ICH-GCP, local regulations, protocol compliance;
- Quality control system which can identify issues on time and resolve/prevent them to satisfaction;
- Robust consent process;
- A well-controlled storage and administration of investigational product with adequate documentation;
- Timely and complete AE reporting/subject safety management.

## CONCLUSION

Drug development is being increasingly globalized and an increased number of patients enrolled in studies submitted as part of applications come from all over the world. Hence, there is an expectation that clinical trials are conducted according to the same ethical and data quality requirements. Inspectors are collaborating and sharing work through joint inspections.
